# An IPv6 address fast scanning method based on local domain name association

**DOI:** 10.1038/s41598-025-95680-w

**Published:** 2025-04-04

**Authors:** Yakai Fang, Liancheng Zhang, Luyang Li, Ce Sun, Yi Guo, Hongtao Zhang, Bin Lin, Jichang Wang, Wenhao Xia

**Affiliations:** 1 School of Cyber Science and Engineering, Zhengzhou University, Zhengzhou, 450001 China; 2 Information Engineering University, Zhengzhou, 450001 China; 3 Key Laboratory of Cyberspace Security, Ministry of Education, Zhengzhou, 450001 China; 4 Network Management Center, Zhengzhou University, Zhengzhou, 450001 China

**Keywords:** IPv6 address scanning, Local domain name, DNS-SD, mDNS, Microsoft Windows Browser, DHCPv6, Electrical and electronic engineering, Computer science, Information technology, Software

## Abstract

With the increase of security issues in IPv6 networks, conducting address scanning in IPv6 networks proves beneficial for identifying potential security risks and vulnerabilities. To enhance the privacy of users’ IPv6 addresses, mainstream OS (Operating System) nodes currently employ randomized interface identifiers and temporary IPv6 addresses. Additionally, since most existing IPv6 address scanning methods rely on active scanning, which makes current on-link IPv6 address scanning methods face the challenges of incomplete scan results, poor coverage across different OSs, significant impact on network performance, and the inability to promptly detect subsequently joined hosts. To this end, An IPv6 address fast scanning method based on local domain name association (FScan6), which combines active scanning and passive listening, is proposed. The active scanning module targets different OSs using distinct protocols (Browser and DNS-SD) to obtain local domain names of on-link hosts. Meanwhile, the passive listening module monitors traffic to extract local domain names of on-link hosts. Then, it employs mDNS protocol to retrieve IPv6 addresses associated with these local domain names. A typical on-link IPv6 network environment was constructed, comprising 26 versions of Windows, Apple, and Linux OSs, and FScan6 was compared with 9 IPv6 address scanning methods. The experimental results show that FScan6 outperforms existing IPv6 address scanning methods in terms of OS coverage and scanning result completeness. Specifically, regarding OS coverage, FScan6 successfully detected all IPv6 addresses across 26 different OS versions, which outperformed 9 address scanning tools and scripts by a factor of 2.89 times at most. Regarding scanning result completeness, FScan6 identified up to 54 additional IPv6 addresses at most compared to these tools and scripts. Additionally, FScan6 has a minimal impact on network performance, with the packet loss rate induced by the tool consistently remaining at 0%.

## Introduction

With the rapid advancement of emerging technologies such as 5G, IoT, and cloud computing, the number of connected devices worldwide had reached 14.3 billion by 2022, far exceeding the 4.3 billion public addresses available under the IPv4 protocol. In contrast, the IPv6 protocol provides a vast pool of IP addresses, ensuring that each connected device can be assigned a unique address, thereby offering superior solutions for end-to-end connectivity, automated configuration, and mobility^1^.

Consequently, the global shift towards IPv6 has become inevitable. In November 2024, the deployment of IPv6 networks accelerated significantly, with coverage in the Asia Pacific region already reaching 49.61%^2^. In some leading countries, such as China, IPv6 coverage has reached 73.67%, with IPv6 traffic on mobile networks exceeding that of IPv4^3^.

The expansion of IPv6 deployment and the improper handling of IPv6 packets by network devices have significantly heightened the security risks faced by IPv6 networks. For example, threateners can exploit the DAD process of the IPv6-specific address configuration mechanism named SLAAC to launch DoS threats. This has also attracted the attention of a wide range of academics who have prevented DoS threats by improving the security mechanisms in the DAD process^4–6^.

Furthermore, the recently disclosed CVE-2024-38063^7^ vulnerability uses the Windows TCP/IP stack’s mishandling of certain anomalous IPv6 packets to enable the remote execution of malicious code. Therefore, threater traceback and vulnerability remediation within IPv6 networks are essential measures to enhance network security. In this context, IPv6 address scanning serves as a foundational task, playing a critical role in supporting these security efforts.

IPv6 address scanning technologies can provide a list of active hosts within an IPv6 network, enabling network administrators to perform vulnerability scans on these IPv6 addresses, identify potential vulnerabilities, and implement necessary patches to ensure the security and reliability of the network.

However, to resolve IPv4 address exhaustion, the IPv6 address length was extended from 32 bits to 128 bits, resulting in an address space containing approximately ${3.4 \times 10^{38}}$ IPv6 addresses. By 2022, the number of globally connected devices stood at only 14.3 billion, and even if each device were assigned 3 IPv6 addresses, this would amount to only $1.26 \times {{10}^{-28}}$ times of the total IPv6 address space.

Due to this vast address space and the sparse distribution of IPv6 addresses, scanning all possible IPv6 addresses one by one within on-link, commonly used 64-bit prefix, is infeasible. Even with the most advanced traversal-based scanning tools, such as Masscan^8^, which can achieve a maximum packet transmission rate of 10 million pps, it would still take tens of thousands of years to complete a full scan.

Meanwhile, as major OSs vendors increasingly prioritize address privacy, modern OSs typically configure 3 IPv6 addresses (including 2 GUAs and 1 LLA. This development poses new challenges for these simple and effective on-link IPv6 address scanning methods, which are facing the risk of not obtaining the complete IPv6 address configuration of target hosts and being blocked by firewalls.

**Fig. 1:**
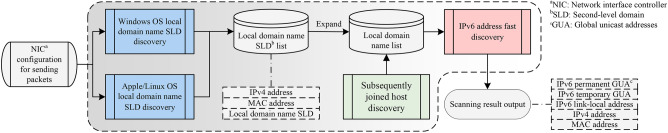
The illustration demonstrates the workflow of FScan6 tool, the FScan6 tool initially employs the local domain name SLD discovery module (including Windows OS local domain name SLD discovery and Apple/Linux OS local domain name SLD discovery module) to discover the SLD of local domain names associated with Windows, Apple, and Linux hosts. These SLDs are then expanded into local domain names. Using this domain name list as input, the IPv6 address discovery module identifies the IPv6 addresses of hosts on-link. Furthermore, to detect the subsequently joined host after the initial scan, FScan6 utilizes the subsequent host discovery module to obtain the domain names of subsequently joined hosts, then employs the IPv6 address discovery module to retrieve the IPv6 addresses of these additional hosts.

To circumvent firewall interception, Hu et al proposed LLMNR6^9^ and LinkScan6^10^. However, these methods are only applicable to IPv4/IPv6 dual-stack networks and only support the acquisition of IPv6 addresses for Windows nodes.

In addition, although there has been some research devoted to obtaining on-link IPv6 addresses, such as DNS-SD based on-link IPv6 address scanning method^11^, most of them are limited to specific network environments and OSs as the above-mentioned LLMNR6 and LinkScan6 methods, and most of them use active scanning methods, which will send a large number of probing packets when there are a large number of on-link hosts and thus affect network performance.

In summary, current on-link IPv6 address scanning methods face the challenges of incomplete scan results, poor coverage across different OSs, significant impact on network performance, and the inability to promptly detect subsequently joined hosts. To address these challenges, this research leverages IPv6 association information and combines active scanning with passive listening proposing an IPv6 address fast scanning method based on local domain name association, which applies to IPv4/IPv6 dual-stack on-link network environment. Furthermore, it designs and implements an automatic IPv6 address scanning tool, named FScan6, which is capable of scanning on-link IPv6 addresses across various OS nodes.

The following are the main objectives and contributions of this paper:

### Objectives

- To address the challenge of poor OS coverage of existing on-link IPv6 address scanning methods.
- To address the challenge of incomplete scan results of existing on-link IPv6 address scanning methods.
- To develop a new IPv6 address scanning method with a combination of active scanning and passive listening and a low impact on network performance.
- To evaluate the effectiveness of the proposed method in coverage of different OSs, the completeness of scanning results , continuous scanning capability, scanning stability, and resource overhead and impact to network.

### Contributions

- This study is the first to combine active scanning with passive listening methods (Sect. “IPv6 address passive listening method based on local domain name association”) to obtain on-link IPv6 addresses. Compared to previous methods, the passive listening method has a reduced impact on network performance, while also achieving the most comprehensive coverage of active IPv6 addresses.
- For the characteristics of on-link nodes, the local domain name discovery method for Windows OS hosts based on service browsing (Sect. “Local domain name active discovery module”), the local domain name discovery method for Apple/Linux OS hosts based on service instance name (Sect. “Local domain name active discovery module”), and the IPv6 address discovery method based on local domain name queries (Sect. “IPv6 address fast discovery module”) are combined to complete the task of scanning on-link IPv6 addresses. Different from previous methods that only targeted the detection of specific OSs, the proposed work can detect the IPv6 addresses of various types of OSs (including Windows, Apple and Linux OS), which has the highest coverage of OS.
- Aiming at the feature that DHCPv6^12^ protocol sends a Solicit packet when a host joins the network, which contains host information, the function of continuous discovery of subsequently joined hosts on-link is designed (Sect. “Subsequently joined host discovery module”).
- Based on the proposed methods, an IPv6 address scanning tool named FScan6 is designed and implemented (the workflow of FScan6 as shown in Fig. 1). Comparative experiments were conducted to validate its effectiveness and superiority (Sect. “Experimental results and analysis”).

Section “Related works” introduces related methods and current research state on IPv6 address scanning. Section “Background” explains the associated information used and the principles by which it can be linked to IPv6 addresses. Section “IPv6 address fast scanning method based on local domain name association” elaborates on the proposed IPv6 address fast scanning method based on local domain name association, following an analysis of the local domain name association characteristics of hosts across various OSs. Section “Experimental results and analysis” validates the effectiveness of the proposed method through comparative experiments. Section “Critical review of the proposed work” evaluates the proposed work from both technical and non-technical perspectives and suggests directions for future research. Finally, the paper concludes with a summary.

In addition, to facilitate reader understanding, Table 1 provides a list of abbreviations used in this paper.

**Table 1:** Abbreviation list.

No.	Abbreviations	Full form	No.	Abbreviations	Full form
1	OS	Operating system	19	mMLD	Nmap scanning script: targets-ipv6-multicast -mld
2	5G	5th-generation mobile communication technology	20	ICMPv6	Internet control message protocol for the IPv6
3	IoT	Internet of things	21	RA	Router advertisement
4	IPv4	Internet protocol version 4	22	MAC	Media access control
5	IPv6	Internet protocol version 6	23	DNS-SD	DNS-based service discovery
6	IP	Internet protocol	24	NBNS	NetBIOS name service
7	DAD	Duplicate address detection	25	LLMNR	Link-local multicast name resolution
8	SLAAC	Stateless address autoconfiguration	26	mDNS	Multicast DNS
9	DoS	Denial of service	27	Zeroconf	Zero configuration networking
10	TCP/IP	Transmission control protocol/internet protocol	28	TLD	Top-level domain
11	pps	Packets per second	29	SLD	Second-level domain
12	MP6	Nmap scanning script: targets-ipv6-multicast-echo	30	SNMP	Simple network management protocol
13	GUA	Global unicast address	31	PTR	Pointer record
14	LLA	Link-local address	32	LAN	Local area network
15	DHCPv6	Dynamic Host configuration protocol for IPv6	33	FQDN	Fully qualified domain name
16	IID	Interface Identifier	34	RQ	Research question
17	EUI-64	64-bit extended unique identifier	35	mSLAAC	Nmap scanning script: targets-ipv6-multicast-slaac
18	IEH	Nmap scanning script: targets-ipv6-multicast-invalid-dst	36	RTT	Round-trip time

## Related works

IPv6 address scanning is a critical technology for identifying active addresses and supporting vulnerability remediation. Due to the vast size of the IPv6 address space, traditional traversal-based scanning methods used in IPv4 networks are no longer feasible. Consequently, researchers have proposed various efficient IPv6 address scanning methods. To systematically explore these methods, this paper categorized IPv6 address scanning methods into 2 types based on whether the scanning node and the target nodes are on the same link: off-link and on-link IPv6 address scanning methods. This section will focus on a detailed analysis of on-link IPv6 address scanning methods.

### Off-link IPv6 address scanning methods

Due to the larger address space of IPv6 compared to IPv4, coupled with the sparse distribution of addresses, off-link IPv6 address scanning methods primarily focus on minimizing the scanning space, thereby enhancing scanning speed.

Current off-link IPv6 address scanning methodologies can be broadly classified into 2 categories: IPv6 address scanning methods based on address statistical information and those based on address prediction.

IPv6 address scanning methods based on address statistical information effectively reduce the IPv6 address search space when the network prefix is known. This is achieved by analyzing common IID generation patterns, such as Low-byte^13^, Embedded-Port^13^, Embedded-IPv4^13^, Wordy^13^, EUI-64^13^, and Modified EUI-64^14^.

IPv6 address scanning methods based on address prediction utilize various methods, such as heuristic algorithms^15–23^ and machine learning algorithms^24–30^, to extract density or structural information from IPv6 seed addresses. By leveraging this information, these methods generate a set of potentially active IPv6 addresses, thereby effectively reducing the address search space and enhancing scanning speed.

However, to enhance user address privacy, many IPv6 nodes have begun employing randomized IIDs, which complicates the ability of address statistical information-based IPv6 address scanning methods to identify active addresses through known IID generation patterns. Furthermore, there are also researchers working on new IPv6 address configuration schemes, such as FDIPA^31^, EPUI-64^32^, and FEUI-64^33^, to reduce the risk of IPv6 address leakage, which makes the heuristic algorithms struggle to uncover the hidden address patterns within randomized IPv6 addresses^34^, resulting in a low prediction accuracy problem for off-link IPv6 address scanning methods.

### On-link IPv6 address scanning methods

Existing on-link IPv6 address scanning methods can be categorized into 2 types based on the underlying protocol stack information: those based on IPv6-only information and those based on IPv4/IPv6 dual-stack association information.

#### Methods based on IPv6-only information

These methods involve sending various types of IPv6 probe packets to get responses from IPv6 hosts, prompting them to feedback on their configured IPv6 addresses to the scanner, thereby completing the detection of IPv6 addresses.

Current on-link IPv6 address scanning methods based on IPv6-only information primarily include MP6^35^ scanning, IEH^36^ scanning, mSLAAC^37^ scanning, and mMLD^38^ scanning.

These methods involve multicasting different types of ICMPv6 probe packets, such as ICMPv6^39^ echo requests, RA packets, ICMPv6 packets with invalid extension headers, or MLD packets, to the link-local scope all-nodes multicast address ff02::1. By capturing the responses or error packets from on-link hosts, the methods can extract the corresponding IPv6 addresses.

However, with the increasing awareness of address security, the above-mentioned straightforward and effective on-link IPv6 address scanning methods face the risk of being blocked by firewalls or layer 2 switches (such as mSLAAC scanning).

#### Methods based on IPv4/IPv6 dual-stack association information

The IPv4/IPv6 dual-stack technology refers to the capability of network devices or systems to support both IPv4 and IPv6 protocol stacks simultaneously, enabling seamless co-existence and interoperability between the 2 protocols. As a widely adopted transition mechanism, it ensures network compatibility and uninterrupted functionality during the gradual migration to IPv6. According to the survey data by Fioccola et al.^40^, dual-stack technology is the most widely adopted solution in wired and cellular networks. Compared to IPv6-only environments, dual-stack technology introduces an additional threat surface through the IPv4 protocol stack. Existing methods enable relatively straightforward access to information within IPv4 networks.

Based on the characteristic of easy information accessibility in IPv4 networks, methods based on IPv4/IPv6 dual-stack association information innovatively leverage the shared information from the IPv4/IPv6 protocol stack, named IPv4/IPv6 dual-stack association information, to perform IPv6 address scanning.

Initially, active hosts are identified within the IPv4 network and obtained their IPv4/IPv6 dual-stack association information, such as hostnames and MAC addresses. Subsequently, employing the relevant protocols to parse these IPv4/IPv6 dual-stack association information and obtain the corresponding IPv6 addresses for those hosts.

Current IPv6 address scanning methods based on IPv4/IPv6 dual-stack association information primarily include the on-link IPv6 address scanning methods based on hostname query, such as LLMNR6^9^ and LinkScan6^10^, as well as that based on the DNS-SD^41^ protocol.

The on-link IPv6 address scanning methods based on hostname query identify active Windows hosts’ hostnames within the IPv4 network environment using the NBNS protocol. Subsequently, it employs the LLMNR^42^ protocol or the mDNS^43^ protocol to derive the corresponding IPv6 addresses from these identified hostnames.

The on-link IPv6 address scanning method based on DNS-SD^11^ retrieves all service records utilizing the DNS-SD protocol from active hosts in an IPv4 network environment. It then obtains the hostnames of these on-link hosts. Finally, it queries the AAAA records associated with the hostnames to acquire the IPv6 addresses of the dual-stack hosts.

#### Limitations of existing methods

Through analysis, existing on-link IPv6 address scanning methods primarily face the following issues:

(1) *Incomplete scanning results.* IPv6 nodes exhibit a characteristic that they have multiple IPv6 addresses, meaning that a single IPv6 node typically possesses a LLA and 2 GUAs. However, current on-link IPv6 address scanning methods are only able to detect a limited number of IPv6 addresses, which means most on-link IPv6 address scanning methods can only scan at most its LLA and 1 temporary IPv6 GUA when scanning the mainstream OS nodes, resulting in a lack of completeness in the IPv6 address scanning results.

(2) *The OS coverage of on-link IPv6 address scanning methods is relatively low.* Most existing on-link IPv6 address scanning methods are designed for specific network environments and OSs. For example, LLMNR6 and LinkScan6 can only scan the IPv6 addresses of Windows OSs. Consequently, due to variations in the detailed implementations of protocols across different OSs, current on-link IPv6 address scanning methods may fail to obtain the desired information when applied to non-target OSs. As a result, these methods are not easily used in address scanning tasks for additional OSs, leading to the issue of limited OS coverage.

(3) *There is a lack of scanning methods that have minimal impact on network performance.* Most existing on-link IPv6 address scanning methods employ active scanning methods. While these methods can quickly and accurately obtain the IPv6 addresses of corresponding nodes, they generate a substantial volume of packets when numerous on-link hosts. This can lead to increased network latency and negatively impact overall network performance, thereby highlighting the issue of significant effects on network efficiency.

(4) *Current on-link IPv6 address scanning methods lack persistent scanning methods.* Existing on-link IPv6 address scanning methods often focus solely on scanning the IPv6 addresses of active on-link hosts at a specific moment, lacking timely and long-term monitoring of hosts that subsequently join the link. This limitation prevents the timely detection of IPv6 address configurations for active hosts that join after the scanning process has concluded, highlighting the issue of insufficient persistent scanning methods.

## Background

This section primarily discusses the principles underlying the acquisition of association information and its correlation to IPv6 addresses.

### Local domain name

In Zeroconf technology, on-link hosts can discover services provided by other on-link hosts without relying on a DNS server. In its concrete implementation, Bonjour technology employs service instance names to record services on-link, formatted as <instance>.<service>.<transport>.local. For example, ‘HostName._rdlink._tcp.local’ is a typical service instance name, where ‘HostName’ refers to the instance name of the host providing the service, ‘_rdlink’ indicates the service provided, and ‘_tcp’ denotes the transport protocol used by the service.

**Fig. 2:**
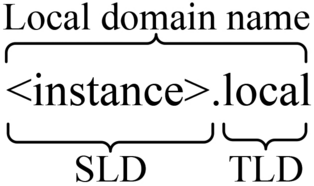
Local domain name structure.

To access the desired service, it is still necessary to obtain the IP address corresponding to the host providing the service. Thus, Bonjour mimics the DNS protocol by assigning a unique domain name within on-link to each active host, whose structure is illustrated in Fig. 2. This domain name consists of the instance name of the host providing the service followed by the TLD ‘local’, enabling the unique identification of a on-link host and facilitating the association of the domain name with the corresponding address configuration.

In this paper, the domain name mentioned above is referred to as the local domain name. As shown in Fig. 2, only the SLD varies between different hosts, meaning that acquiring the SLD is sufficient to construct the complete local domain name.

## IPv6 address fast scanning method based on local domain name association

This section addresses the issues of low completeness of IPv6 address scanning results, significant impact on network performance, and limited OS coverage in existing on-link IPv6 address scanning methods. Building on the analysis of the association information present in mainstream OSs, a novel IPv6 address fast scanning method based on local domain name association is proposed.

### Characterization analysis of local domain name associations for mainstream OSs

Following the analysis of the challenges faced by on-link IPv6 address scanning methods in Section ‘On-link IPv6 address scanning methods’, this section further examines current mainstream OS nodes. The analysis reveals that all mainstream OS nodes now have the IPv6 protocol stack enabled by default. Additionally, to simplify the network configuration process, most of these systems have configured local domain names to facilitate the automatic discovery of services on-link.

(1) *Most mainstream OSs are configured with local domain names to facilitate service discovery automatically.* The local domain name is an important basis for identifying the services provided by different on-link hosts, so it is unique on-link. Furthermore, to access the services offered by a particular host, the local domain name is typically resolvable to the host’s address configuration. The local domain name can be feasibly used to discover the address configuration of the corresponding host.

(2) *Utilizing local domain name association can effectively reduce the scanning space for on-link IPv6 address scanning methods.* The association of local domain names begins by utilizing relevant protocols to discover the services offered by active on-link hosts, from which the local domain names are extracted. Subsequently, these local domain names are used to retrieve their corresponding IPv6 addresses. This method cleverly transforms the large address space scanning task in the on-link IPv6 network environment into a focused scanning task targeting only the small address space of active on-link hosts. This effectively reduces the address space to be scanned and significantly enhances the efficiency of on-link IPv6 address scanning.

**Fig. 3:**
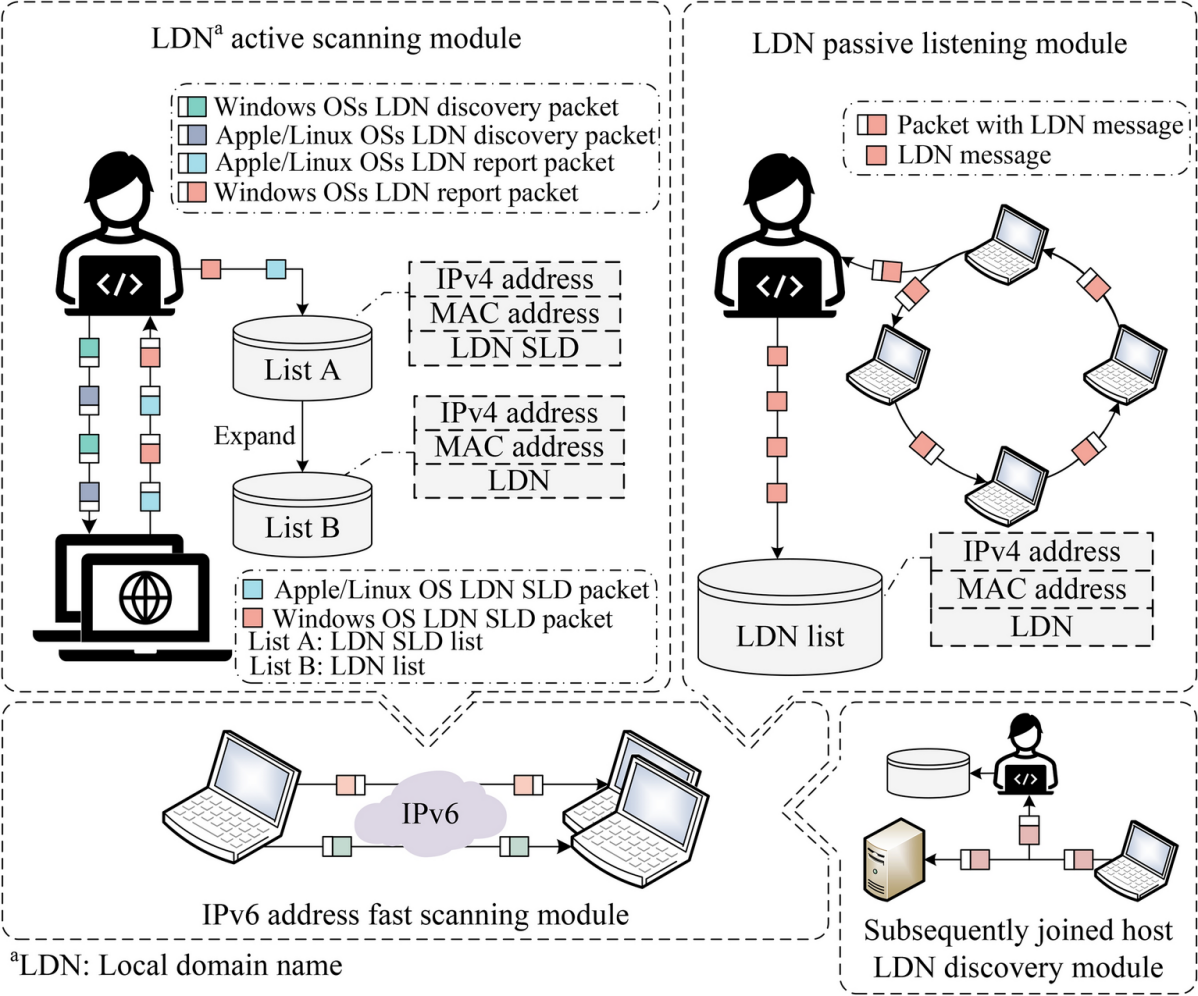
Schema of IPv6 address fast scanning method based on local domain name association.

Based on the analysis above, and to address the issues of low completeness of scanning results, significant impact on network performance, and limited OS coverage in existing on-link IPv6 address scanning methods, this paper investigates the associations and shared information among various mainstream OS nodes. By leveraging the protocol support characteristics specific to different OSs, this paper employs distinct protocols to obtain their corresponding local domain names. Utilizing the property that local domain names can be associated with host address configurations, a new method aimed at enhancing the completeness of IPv6 address scanning results while supporting both passive and active address scanning is proposed. The overall process is illustrated in Fig. 3.

As shown in Fig. 3, this proposed method is primarily divided into 2 categories: IPv6 address active scanning method based on local domain name association and IPv6 address passive listening method based on local domain name association.

*IPv6 address active scanning method based on local domain name association:* This method includes the “LDN active scanning module” and the “IPv6 address fast discovery module” in Fig. 3. It first uses the “LDN active discovery module” to identify the local domain names list of active on-link hosts, then the “IPv6 address fast discovery module” is used to get the IPv6 address configuration information by using local domain name list obtained from the last module.

*IPv6 address passive listening method based on local domain name association:* This method includes the “LDN passive listening module”, the “Subsequently joined host LDN discovery module” and the “IPv6 address fast discovery module” in Fig. 3. It first uses the “Local domain name passive discovery module” to monitor the packets circulating on-link. From these packets, this module extracts the local domain names of active on-link hosts, and then the IPv6 address fast discovery module is used to obtain the corresponding IPv6 addresses by using the local domain name list obtained from the last modules. After this, the passive listening method can also use the “Subsequently joined host discovery module” to discover the hosts’ local domain name newly joined the on-link, and use the “IPv6 address fast discovery module” to discover their IPv6 addresses.

### IPv6 address active scanning method based on local domain name association

**Fig. 4:**
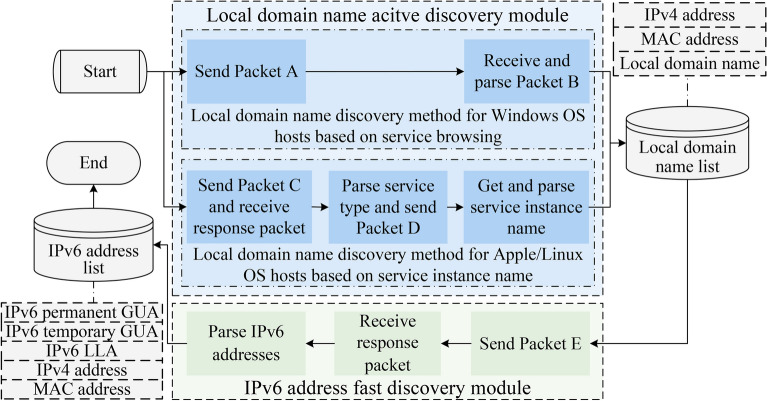
The illustration demonstrates the flowchart of IPv6 address active scanning method based on local domain name association, where **Packet A** refers to the request announcement packet in Microsoft Windows Browser protocol, **Packet B** refers to the host announcement packet in Browser protocol, **Packet C** refers to the service type enumeration request packet, **Packet D** refers to the service instance name query packet, and **Packet E** refers to the mDNS query packet.

The fundamental principle of IPv6 address active scanning method involves sending probing packets to target hosts, prompting the target hosts to respond with packets from which information related to local domain names can be extracted. Consequently, the IPv6 address active scanning method based on local domain name association can utilize various protocols to discover host identification information for different OSs, which is the SLD of the local domain name. Subsequently, the SLD is expanded into the local domain name. Then the protocols capable of resolving local domain names are used to discover the corresponding IPv6 addresses. The specific process is illustrated in Fig. 4.

#### Local domain name active discovery module

The protocols currently available for obtaining the SLD of the local domain name primarily include the SNMP^44^ and the DNS protocol.

The SNMP is a standard protocol used for managing network nodes within IP networks, enabling network administrators to monitor, configure, and manage devices in the network. However, due to various security vulnerabilities associated with SNMP, it is not enabled by default in current mainstream OSs.

The DNS protocol’s PTR records can be used to resolve IP addresses to globally unique domain names. However, in the on-link context, these records must be configured by a network administrator. If there is no DNS server on-link or if the administrator has not configured the PTR records for local hosts, it becomes impossible to discover the local domain names of on-link hosts using this method. Through experimentation and analysis, it has been observed that the aforementioned local domain name discovery protocols all face the issue of being unable to effectively discover local domain names if they are not correctly configured. Consequently, these protocols cannot be utilized for the discovery of the SLD for on-link hosts.

The Browser protocol and the DNS-SD protocol are respectively implemented in Windows and Apple OSs, and are enabled by default in their corresponding environments. Moreover, these 2 protocols support the discovery of local domain name SLD across multiple versions of OSs. The Browser protocol is available in current mainstream Windows systems, while the DNS-SD protocol is supported in macOS 10.14.5, iOS 11.4, and iPadOS 11.4 and later versions.

1. Local domain name discovery method for Windows OS hosts based on service browsing

In the Server Message Block protocol, a computer must be aware of the list of available resources on the network before it can access them-a process known as browsing. However, if the network resources are rediscovered via broadcasting each time, it would result in significant bandwidth consumption and time inefficiency.

To address the issue above, the Browser protocol was introduced as an auxiliary protocol to the Server Message Block protocol. It allows on-link hosts to elect a Master Browser, which maintains an up-to-date list of network resources. On-link hosts can also actively send request announcement packets to prompt other hosts on the network to announce their presence via host announcement packets. The host announcement packet contains the NetBIOS name, which uniquely identifies a on-link node. In Windows systems, the NetBIOS name corresponds to the SLD portion of the local domain name.

Based on this principle, the method constructs and sends a request announcement packet with the destination MAC address set to the broadcast address ff:ff:ff:ff:ff:ff and the destination IPv4 address set to the LAN broadcast address. Upon receiving this packet, on-link hosts will respond by sending a host announcement packet to the scanner within 30 seconds. From these responses, the scanner can extract the IPv4 addresses and NetBIOS names of active on-link hosts, thereby obtaining their corresponding local domain names. The detailed implementation is outlined in Algorithm 1.

Analysis reveals that Algorithm 1 exhibits both time and space complexity of *O*(*n*), where *n* denotes the number of active hosts on-link. Specifically, the time complexity arises primarily from two operations: receiving and parsing response packets (*O*(*n*)) and expanding the SLD list to local domain name list (*O*(*n*)). Meanwhile, the space complexity is determined by the storage requirements for the parsed SLD list from the response packets (*O*(*n*)) and the generated local domain name list (*O*(*n*)).

**Algorithm 1:**
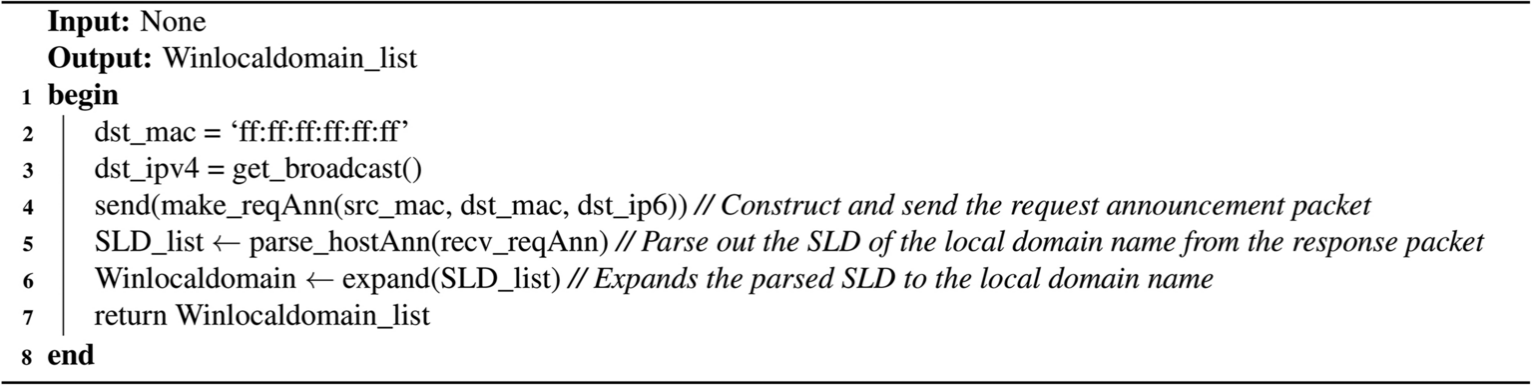
Algorithm for active discovery of local domain on Windows hosts.

(2) Local domain name discovery method for Apple/Linux OS hosts based on service instance name.

**Fig. 5:**
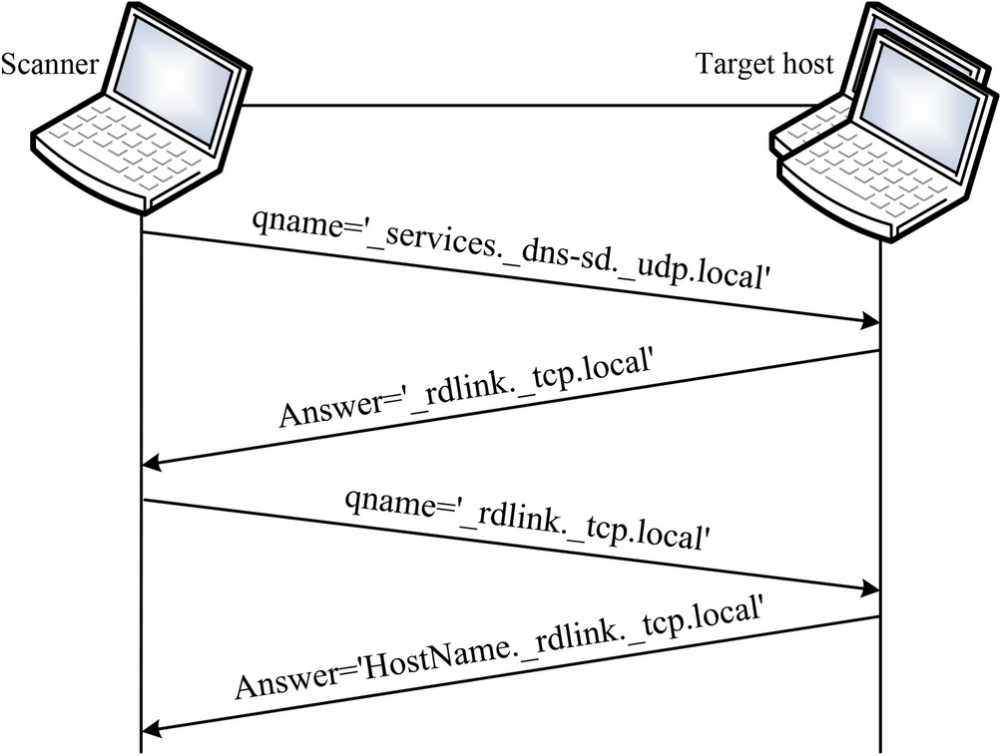
The workflow of DNS-SD protocol.

Bonjour technology is a cross-platform, Zeroconf technology designed to simplify the process of connecting network devices. It allows users to discover and connect to services, such as printers, other computers, and device-based services (like AirPlay or file sharing), within on-link network environment without requiring complex network configurations. Bonjour technology is primarily based on 2 standard network protocols that are extensions built within the standard DNS framework: the DNS-SD protocol and the mDNS protocol.

DNS-SD protocol in Bonjour technology mainly performs the service discovery function. It allows automatic discovery of services in the network without pre-configuration, which workflow is shown as Fig. 5.

According to Fig. 5, the method first sends a service type enumeration request packet with the query name ‘_services._dns-sd._udp.local’ and the type of PTR record to the IPv4 LAN multicast address 224.0.0.251, which induces the active on-link hosts send their own provided service type to the scanner. Next, a DNS-SD query packet with its service type as the query name is sent to the corresponding host. Finally, listens on port 5353 and receives the response packet from those hosts, and parses the <instance> part of the service instance names and the IPv4 addresses of the active on-link hosts in the packet. Algorithm 2 outlines the specific implementation.

Analysis indicates that Algorithm 2 has both time and space complexities of *O*(*n*), where *n* represents the number of active hosts on-link. The time complexity primarily arises from 2 factors: receiving and parsing service type from response packets (*O*(*n*)) and loop processing the service instance names corresponding to each service type (*O*(*n*)). Similarly, the space complexity is determined by the storage requirements for the service type list (*O*(*n*)), the SLD list (*O*(*n*)), and the final generated local domain name list (*O*(*n*)).

**Algorithm 2:**
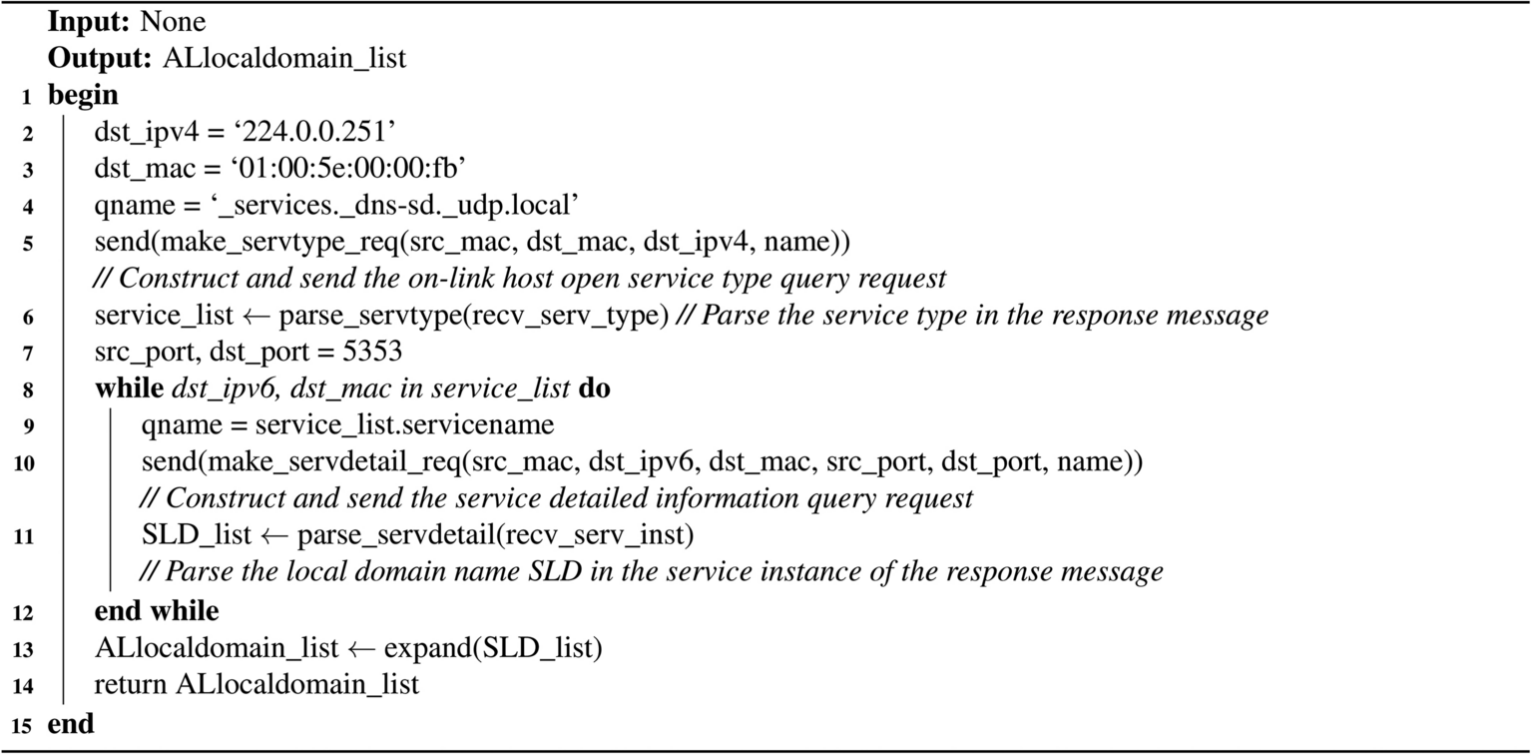
Algorithm for active discovery of local domain name on Apple/Linux OS hosts.

#### IPv6 address fast discovery module

mDNS is a technology that enables on-link devices to discover one another through multicast DNS packets. It allows on-link devices to directly use local domain names to obtain IP addresses via multicast, without the need for a central DNS server.

**Algorithm 3:**
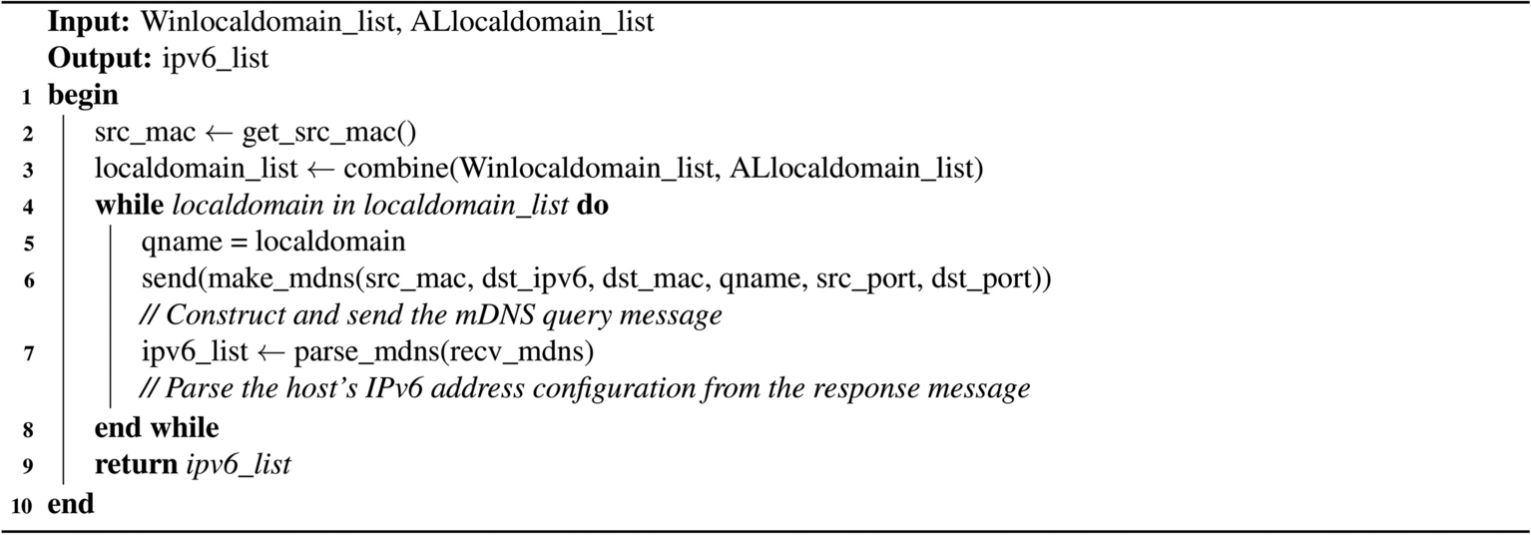
Algorithm for IPv6 address fast discovery.

The mDNS protocol utilizes the local domain names obtained from the DNS-SD protocol as query names to send DNS queries to the target host, prompting the target host to return its address configuration.

According to the above principle, the module takes as input the list of local domains found by the previous two modules, constructs and sends the mDNS IPv6 address probe packet whose destination MAC address is the standard multicast MAC address 01:00:5e:00:00:fb, destination IPv6 address is the mDNS special multicast address ff02::fb, and the query name is the local domain name discovered by the before modules. Next, the active on-link hosts will send their IP configuration information to the scanner after receiving the probe packet, from which its configured IPv6 address information can be resolved. The specific implementation is outlined in Algorithm 3.

Analysis reveals that Algorithm 3 exhibits both time and space complexities of *O*(*n*), where *n* denotes the number of active hosts on-link. Specifically, the time complexity is primarily influenced by two factors: merging the local domain name list (*O*(*n*)), and parsing mDNS query messages (*O*(*n*)). Similarly, the space complexity is determined by the storage requirements for the merged local domain name list (*O*(*n*)), and the final generated IPv6 address list (*O*(*n*)).

### IPv6 address passive listening method based on local domain name association

**Fig. 6:**
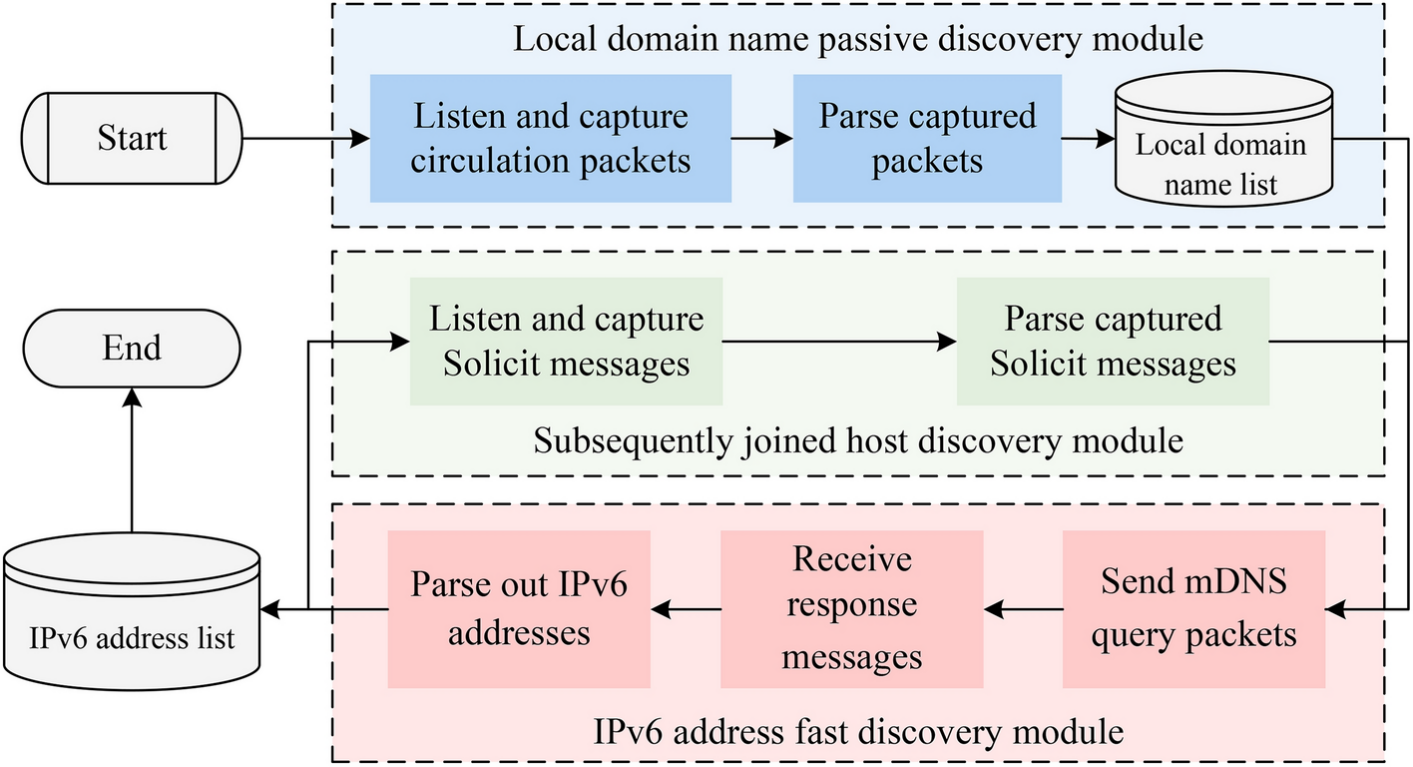
Flowchart of IPv6 address passive listening method based on local domain name association.

Instead of actively sending probe packets, IPv6 address passive listening method listens for packets circulating in the network. Then, it captures eligible packets flowing through the network interface. Finally, it analyses the contents of these packets to obtain useful information. Consequently, the IPv6 address passive listening method based on local domain name association first monitors the packets flowing through the network to identify the local domain name SLD of active on-link hosts. Subsequently, this method expands SLD into the full local domain name. Then the protocols capable of resolving local domain names are employed to discover the corresponding IPv6 addresses. The specific process is illustrated in Fig. 6.

**Fig. 7:**
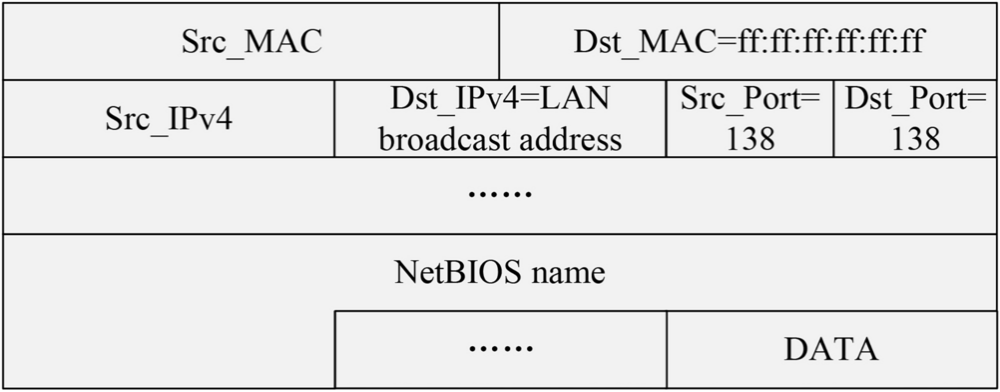
Host announcement packet structure.

**Fig. 8:**
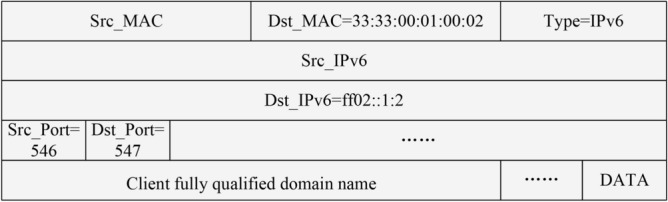
Solicit packet structure.

The Browser protocol requires the active on-link hosts to send a host announcement packet at regular intervals to update the status of their supported services in the list of services stored by the Master Browser. The structure of the host announcement packet is shown in Fig. 7. It uses the IPv4 LAN broadcast address as the destination address, 138 as the destination port, and the broadcast MAC address ff:ff:ff:ff:ff:ff as the destination MAC address. The NetBIOS name option part of the packet contains the NetBIOS name of the target host, which is the SLD of the local domain name.

According to the above principle, the module listens to the packets sent to port 138 whose destination IPv4 address is an IPv4 LAN broadcast address and whose destination MAC address is the broadcast MAC address ff:ff:ff:ff:ff:ff, to parse out the SLD of the local domain name contained therein. Then this module proceeds to expand the SLD into the local domain name corresponding to that host. Finally, the list of local domain names of active on-link hosts is entered into the IPv6 address fast discovery module to discover their corresponding IPv6 addresses.

#### Subsequently joined host discovery module

The DHCPv6 protocol is one of the foundational protocols in IPv6 networks, which is used to automatically assign IPv6 addresses and their network configuration parameters to hosts in an IPv6 network. It is implemented in most different types of OSs. This simplifies network management by eliminating the need for network administrators to manually configure each host’s IPv6 address and other related information.

When a host joins an IPv6 network for the first time, it sends a Solicit packet, which structure is shown in Fig. 8 to the multicast address ff02::1:2 to initialize the address request and to discover the on-link DHCPv6 servers, and the Solicit packet contains a series of information to help the client to express its needs. The Solicit packet uses port 547 as the destination port, ff02::1:2 as the destination IPv6 address, and contains the Client FQDN^45^, which is the local domain name of the host.

Accordingly, this module listens to port 547 to capture DHCPv6 Solicit packets with destination address ff02::1:2. Then this module resolves the link-local IPv6 address of the subsequently joined hosts and their corresponding local domain names from these packets. Finally, the above information is passed as input to the IPv6 address fast discovery module to discover the corresponding IPv6 addresses of the subsequently joined hosts.

## Experimental results and analysis

The FScan6 tool is implemented based on Python and the packet processing tool Scapy^46^. In this section, it first present details of the test environment (Section “Experimental environment and methods”) and then organizes our experimental results around the 5 key RQs. These research questions are listed below:

**(RQ1)**: Can the proposed FScan6 method effectively detect IPv6 addresses of different types of OSs? (Coverage of different OSs)

**(RQ2)**: Can the proposed FScan6 method scan the most complete IPv6 address when scanning a single host? (Completeness of scanning results)

**(RQ3)**: Can the proposed FScan6 method scan for subsequently joined hosts? (Continuous scanning capability)

**(RQ4)**: Can the proposed FScan6 method ensuring result completeness across different network environments? (Scanning stability)

**(RQ5)**: What are the system resource overhead requirements of the proposed FScan6 method, and how does it impact network performance? (Scan overhead and impact on network performance analysis)

### Experimental environment and methods

**Fig. 9:**
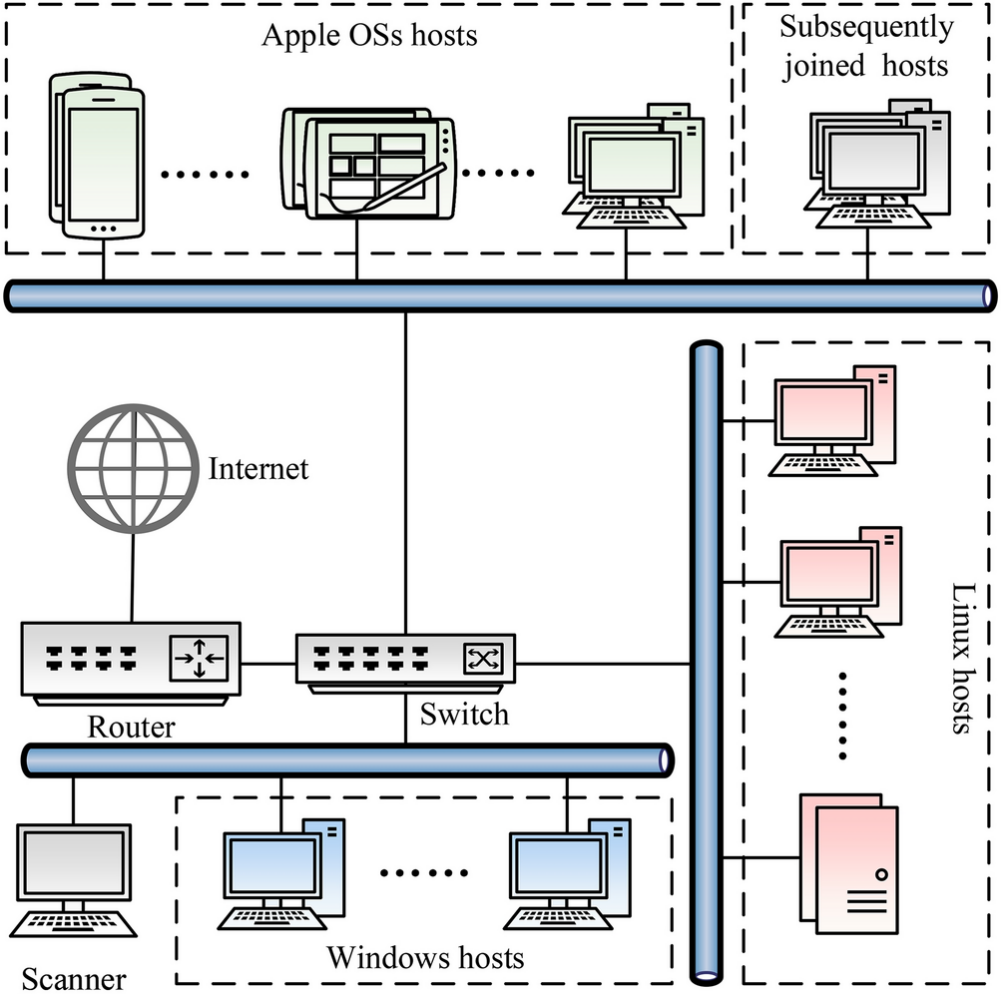
IPv6 network topology for comparative experiments.

*Experimental environment setting* In this paper, an experimental environment is built as shown in Fig. 9, which contains 9 desktop and server versions of Windows nodes, 10 desktop and mobile versions of Apple OS nodes, and 7 desktop and server versions of Linux nodes, the OS versions and types of the nodes involved in the experiment are detailed in Table 2. The scanner is equipped with an Intel(R) Core(TM) i9-14900HX CPU 2.20 GHz and 32GB RAM.

**Table 2:** The list of nodes involved in the experiment.

OS type	OS versions	OS type	OS versions	OS type	OS versions
Apple	macOS 10	Windows	Windows XP	Linux	Ubuntu 20.04
	macOS 11		Windows 7		Ubuntu 21.04
	macOS 12		Windows 8		Ubuntu 22.04
	macOS 13		Windows 10		Ubuntu 23.04
	iOS 15		Windows 11		Ubuntu Server 20.04
	iOS 16		Windows Server 2012		Ubuntu Server 22.04
	iOS 17		Windows Server 2016		Ubuntu Server 23.10
	iPadOS 15		Windows Server 2019		
	iPadOS 16		Windows Server 2025		
	iPadOS 17				

*Research methodology* Firstly, for **RQ1**, a typical on-link IPv6 network environment is constructed to evaluate the detection capability of the FScan6 tool for different types of OSs. For **RQ2**, the above on-link IPv6 network environment is used as an experimental environment and the number of scanned IPv6 addresses as a percentage of the total IPv6 addresses (Scanning Completeness) as an evaluation indicator to evaluate the scanning completeness of FScan6 for different types of OS nodes. For **RQ3**, the scanner is first made to start running the subsequently joined host scanning module. Then, new hosts are added to the existing on-link IPv6 network environment and observe whether the scanner can scan the IPv6 addresses of the subsequently joined hosts. For **RQ4**, this study simulates network environments with different bandwidths and assesses the scanning stability of the FScan6 tool by measuring the number of OS versions and the number of IPv6 addresses it scans in these network environments. For **RQ5**, this study monitors CPU usage and memory usage when running the FScan6 tool and other comparison methods to evaluate the resource consumption generated by the FScan6 tool. Moreover, this study also runs the FScan6 tool under different network loads and measures the RTT latency and packet loss rate of normal network traffic through ICMPv6 echo requests to quantify the impact of the FScan6 tool on network performance.

### Experimental results

In this section, the scanning capabilities of FScan6 are evaluated from 5 key perspectives: coverage of different OSs (corresponding to **RQ1**), the completeness of scanning results (corresponding to **RQ2**), continuous scanning capability (corresponding to **RQ3**), scanning stability (corresponding to **RQ4**), and resource overhead and impact to network (corresponding to **RQ5**).

**Fig. 10:**
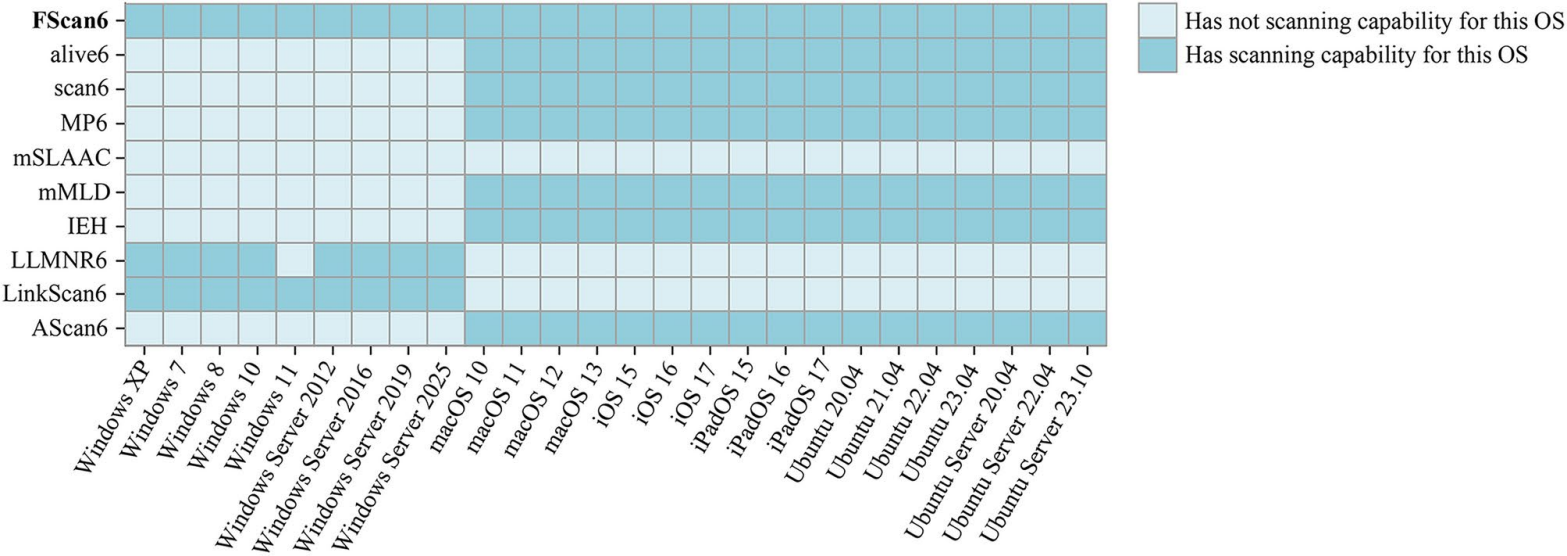
Experimental results of coverage of different OSs.

#### Coverage of different OSs (to answer RQ1)

In this paper, it use FScan6, the alive6 address scanning tool in the IPv6 network penetration testing toolkit THC-IPv6^47^, the scan6 tool in IPv6Toolkit^48^, 4 IPv6 address scanning scripts in Nmap: MP6^35^, mSLAAC^37^, mMLD^38^, and IEH^36^ scanning scripts as well as 3 on-link IPv6 address scanning tools based on association information: LLMNR6, LinkScan6 and AScan6^49^. Performs address scanning on the hosts in Fig. 9. The results are shown in Fig. 10.

According to the experimental results in Fig. 10, the MP6, IEH, and mMLD scanning scripts, alive6, scan6 and AScan6 can scan the IPv6 addresses of OSs other than Windows, which is mainly because to protect the system security the Windows firewall blocks the scanning packets sent out by the above-mentioned scripts by default, which makes it impossible for the target hosts to respond. For all types of OSs, the mSLAAC scripts are unable to scan for IPv6 addresses. The main reason is that the experimental device is placed behind a layer 2 switch with the ICMPv6-based access control lists, which will only receive ICMPv6 RA packets from directly connected routers, and block the RA packets from untrusted ports.

LLMNR6 and LinkScan6 are only able to scan IPv6 addresses on Windows OSs, mainly because they use the NBNS protocol for hostname discovery of active on-link hosts, which is only implemented on Windows systems.

FScan6 shows the most comprehensive scanning capability for different OSs. It can scan the IPv6 addresses of all 26 different versions and types of OSs. This is because FScan6 uses different protocols for different OSs to obtain their corresponding local domain names. This avoids the situation that a certain protocol is only implemented in a certain OS, and achieves the matching of different types of OSs.

#### Completeness of scanning results (to answer RQ2)

**Table 3:** The table demonstrates the detailed scanning results for various OS versions, where GUA1 refers to the IPv6 permanent GUA, and GUA2 refers to the IPv6 temporary GUA.

**OS versions**	Scanning tools									
	FScan6	alive6	scan6	MP6	mSLAAC	mMLD	IEH	LinkScan6	LLMNR6	AScan6
Windows XP	GUA1/GUA2/LLA	–	–	–	–	–	–	GUA1/GUA2/LLA	GUA1/GUA2/LLA	–
Windows 7	GUA1/GUA2/LLA	–	–	–	–	–	–	GUA1/GUA2/LLA	GUA1/GUA2/LLA	–
Windows 8	GUA1/GUA2/LLA	–	–	–	–	–	–	GUA1/GUA2/LLA	GUA1/GUA2/LLA	–
Windows 10	GUA1/GUA2/LLA	–	–	–	–	–	–	GUA1/GUA2/LLA	GUA1/GUA2/LLA	–
Windows 11	GUA1/GUA2/LLA	–	–	–	–	–	–	–	GUA1/GUA2/LLA	–
Windows server 2012	GUA1/LLA	–	–	–	–	–	–	GUA1/LLA	GUA1/LLA	–
Windows server 2016	GUA1/LLA	–	–	–	–	–	–	GUA1/LLA	GUA1/LLA	–
Windows server 2019	GUA1/LLA	–	–	–	–	–	–	GUA1/LLA	GUA1/LLA	–
Windows Server 2025	GUA1/LLA	–	–	–	–	–	–	GUA1/LLA	GUA1/LLA	–
macOS 10	GUA1/GUA2/LLA	LLA	GUA2/LLA	GUA2/LLA	–	LLA	GUA2/LLA	–	–	GUA1/GUA2/LLA
macOS 11	GUA1/GUA2/LLA	LLA	GUA2/LLA	GUA2/LLA	–	LLA	GUA2/LLA	–	–	GUA1/GUA2/LLA
macOS 12	GUA1/GUA2/LLA	LLA	GUA2/LLA	GUA2/LLA	–	LLA	GUA2/LLA	–	–	GUA1/GUA2/LLA
macOS 13	GUA1/GUA2/LLA	LLA	GUA2/LLA	GUA2/LLA	–	LLA	GUA2/LLA	–	–	GUA1/GUA2/LLA
iOS 15	GUA1/GUA2/LLA	LLA	GUA2/LLA	GUA2/LLA	–	LLA	GUA2/LLA	–	–	GUA1/GUA2/LLA
iOS 16	GUA1/GUA2/LLA	LLA	GUA2/LLA	GUA2/LLA	–	LLA	GUA2/LLA	–	–	GUA1/GUA2/LLA
iOS 17	GUA1/GUA2/LLA	LLA	GUA2/LLA	GUA2/LLA	–	LLA	GUA2/LLA	–	–	GUA1/GUA2/LLA
iPadOS 15	GUA1/GUA2/LLA	LLA	GUA2/LLA	GUA2/LLA	–	LLA	GUA2/LLA	–	–	GUA1/GUA2/LLA
iPadOS 16	GUA1/GUA2/LLA	LLA	GUA2/LLA	GUA2/LLA	–	LLA	GUA2/LLA	–	–	GUA1/GUA2/LLA
iPadOS 17	GUA1/GUA2/LLA	LLA	GUA2/LLA	GUA2/LLA	–	LLA	GUA2/LLA	–	–	GUA1/GUA2/LLA
Ubuntu 20.04	GUA1/GUA2/LLA	LLA	GUA2/LLA	GUA2/LLA	–	LLA	GUA2/LLA	–	–	GUA1/GUA2/LLA
Ubuntu 21.04	GUA1/GUA2/LLA	LLA	GUA2/LLA	GUA2/LLA	–	LLA	GUA2/LLA	–	–	GUA1/GUA2/LLA
Ubuntu 22.04	GUA1/GUA2/LLA	LLA	GUA2/LLA	GUA2/LLA	–	LLA	GUA2/LLA	–	–	GUA1/GUA2/LLA
Ubuntu 23.04	GUA1/GUA2/LLA	LLA	GUA1/LLA	GUA1/LLA	–	LLA	GUA1/LLA	–	–	GUA1/GUA2/LLA
Ubuntu Server 20.04	GUA1/LLA	LLA	GUA1/LLA	GUA1/LLA	–	LLA	GUA1/LLA	–	–	GUA1/LLA
Ubuntu Server 22.04	GUA1/LLA	LLA	GUA1/LLA	GUA1/LLA	–	LLA	GUA1/LLA	–	–	GUA1/LLA
Ubuntu Server 23.10	GUA1/LLA	LLA	GUA1/LLA	GUA1/LLA	–	LLA	GUA1/LLA	–	–	GUA1/LLA

**Table 4:** Experimental results of scanning completeness.

Scanning tools	Windows versions	Apple versions	Linux versions	Scanned IPv6 addresses	Scanning speed (s)	Scanning completeness (%)
**FScan6**	**9**	**10**	**7**	**71**	36.02	**100**
alive6	0	10	7	17	5.74	23.94
scan6	0	10	7	34	4.25	47.89
MP6	0	10	7	34	3.10	47.89
mSLAAC	0	0	0	0	3.82	0
mMLD	0	10	7	17	13.38	23.94
IEH	0	10	7	34	**2.66**	47.89
LinkScan6	8	0	0	20	7.39	28.17
LLMNR6	9	0	0	23	7.34	32.39
AScan6	0	10	7	48	12.22	67.61

Significant values are in [bold].

To evaluate the scanning completeness of the proposed method, the scanners run the above 9 scanning tools separately in a typical on-link IPv6 network environment shown in Fig. 9, and record the number of addresses scanned by each scanning tool for a single OS. The scanning completeness is used to measure the experimental results. The calculation method is shown below, where the total number of IPv6 addresses in this experiment is constant at 71. The results of the experiment are shown in Tables 3 and 4, where Table 3 describes the detailed results of the experiment, Table 4 describes the scanning completeness of each of the participating tools.

1 $$\begin{aligned} Scanning\; Completeness=\frac{Scanned\; IPv6\; addresses}{Total\; IPv6\; addresses} \times 100\% \end{aligned}$$

As shown in Table 3, for Windows OS nodes, FScan6, LLMNR6, and LinkScan6 can scan the most complete IPv6 addresses (including all GUAs and LLA). The main reason of LLMNR6 can not obtain the IPv6 addresses of Windows 11 is that although Windows 11 supports the LLMNR protocol, its firewall has blocked the protocol.

For Apple and Linux OSs, only FScan6 and AScan6 can detect all IPv6 addresses of all versions of Apple OS and Linux OS nodes, while MP6 and IEH scanning scripts can only detect 1 IPv6 temporary GUA and 1 IPv6 LLA of the above 2 types of OS. The MP6 and IEH scanning scripts send probe packets with all the IPv6 addresses of the scanner as the source IPv6 addresses, and the target hosts will send response packets with the corresponding IPv6 addresses after receiving the probe packets. To protect the host’s address privacy, the target host usually does not respond to the probe packets sent by the 2 address scanning scripts with a permanent IPv6 GUA as the source IPv6 address. The mMLD address scanning script only sends scanning scripts with LLAs, so it can only scan the IPv6 LLAs of the above 2 types of OSs.

On the other hand, alive6 can only detect 1 temporary IPv6 address for Linux and Apple OSs, while scan6 can detect 1 IPv6 temporary GUA and 1 LLA for Linux and Apple OSs. The alive6 and scan6 methods combine the MP6 and IEH methods. The alive6 only sends probe packets with the scanner’s IPv6 temporary GUA as the source address, while scan6 sends probe packets with the scanner’s IPv6 temporary GUA and the LLA as the source address. Therefore, the target host will respond to the alive6 method with the IPv6 temporary GUA as the source address and respond to the scan6 method with the IPv6 temporary GUA and the LLA as the source address.

As shown in Table 4, in terms of scanning completeness, FScan6 achieves a 100% completion rate in scanning IPv6 addresses across 26 different operating system versions, demonstrating the highest address scanning comprehensiveness. Its performance surpasses that of the alive6 and mMLD tools by a factor of 4.17, the LinkScan6 tool by 3.55 times, the LLMNR tool by 3.09 times, and the scan6, MP6, and IEH tools by 2.09 times. Notably, it also outperforms the AScan6 tool by 1.48 times.

However, in terms of scanning speed, FScan6 exhibits the highest time consumption. This is not only due to its involvement in multiple stages of packet transmission, reception, and parsing but also because, during the local domain name probing phase for Windows hosts, the target host’s response packets are randomly sent within 30 seconds after receiving the probe packet. Consequently, FScan6 waits for 30 seconds to ensure that no response packet containing local domain names is missed.

In summary, FScan6 can scan all IPv6 addresses of all 26 different versions of different types of OSs and can obtain the most complete IPv6 addresses within minute-level time consumption.

#### Continuous scanning capability (to answer RQ3)

To evaluate the effectiveness of the subsequently joined host discovery module of the proposed method, firstly, this experiment runs the subsequently joined host discovery module of the FScan6 tool and then adds new hosts to the existing network environment with the following OS types: Windows XP, Windows 7, Windows 8, Windows 10, and CentOS 8. The results of the scanning are shown in Table 5.

**Table 5:** Experimental results of continuous scanning capability.

Scanning tool	Windows XP	Windows 7	Windows 8	Windows 10	CentOS 8
FScan6	GUA1/GUA2/LLA	GUA1/GUA2/LLA	GUA1/GUA2/LLA	GUA1/GUA2/LLA	GUA1/GUA2/LLA

According to Table 5, FScan6 can effectively Windows and some Linux systems subsequently join all IPv6 addresses of the host. As for the Apple OSs, due to the difference in the implementation details of the DHCPv6 protocol between this type of OS and the above 2 types of OSs, the Solicit packet sent when joining the network for the first time does not contain the Client FQDN of the host, and therefore the local domain name of the host of the Apple OSs cannot be obtained, which in turn cannot be obtained for the IPv6 address of the host.

#### Scanning stability (to answer RQ4)

To evaluate the scanning stability of the FScan6 tool, a set of comparative experiments was designed. Specifically, traffic control tools were used to simulate eight different network environments with varying bandwidths, burst sizes, and latencies, as outlined in Table 6. The FScan6 tool was run in each environment, and the number of detected OS versions as well as the completeness of the scanning results were measured. The experimental results are presented in Table 7.

**Table 6:** The table demonstrates the detailed network environment settings used in the experiment to assess the impact of the FScan6 tool on network performance, where the Burst size refers to the maximum amount of data permitted within a short time frame, the Latency indicates the maximum packet wait time allowed in the queue.

No.	Data rate (Mbps)	Burst (KB)	Latency (ms)
1	1	2	100
2	2	5	100
3	5	15	80
4	10	20	80
5	50	100	60
6	100	200	50
7	200	400	40
8	500	1000	30

**Table 7:** Coverage of different OSs and scanning completeness in different network environment.

Network environment No.	Number of scanned OS versions	Scanning completeness (%)
1	26	100
2	26	100
3	26	100
4	26	100
5	26	100
6	26	100
7	26	100
8	26	100

As experimental results are shown in Table 7, the FScan6 tool performed exceptionally well across all test scenarios. In every network environment, FScan6 was able to detect all IPv6 addresses for the 26 OS versions. This outstanding performance can be largely attributed to the FScan6’s use of multicast by FScan6 for scanning tasks. The multicast mechanism enables FScan6 to complete IPv6 address configuration scans on-link hosts with minimal packet transmission, significantly reducing the likelihood of packet loss.

#### Scan overhead and impact on network performance analysis (to answer RQ5)

To assess the detection performance overhead of FScan6, this section conducts tests from two perspectives: operational resource overhead and impact on network performance.

1. Running overhead of FScan6

**Fig. 11:**
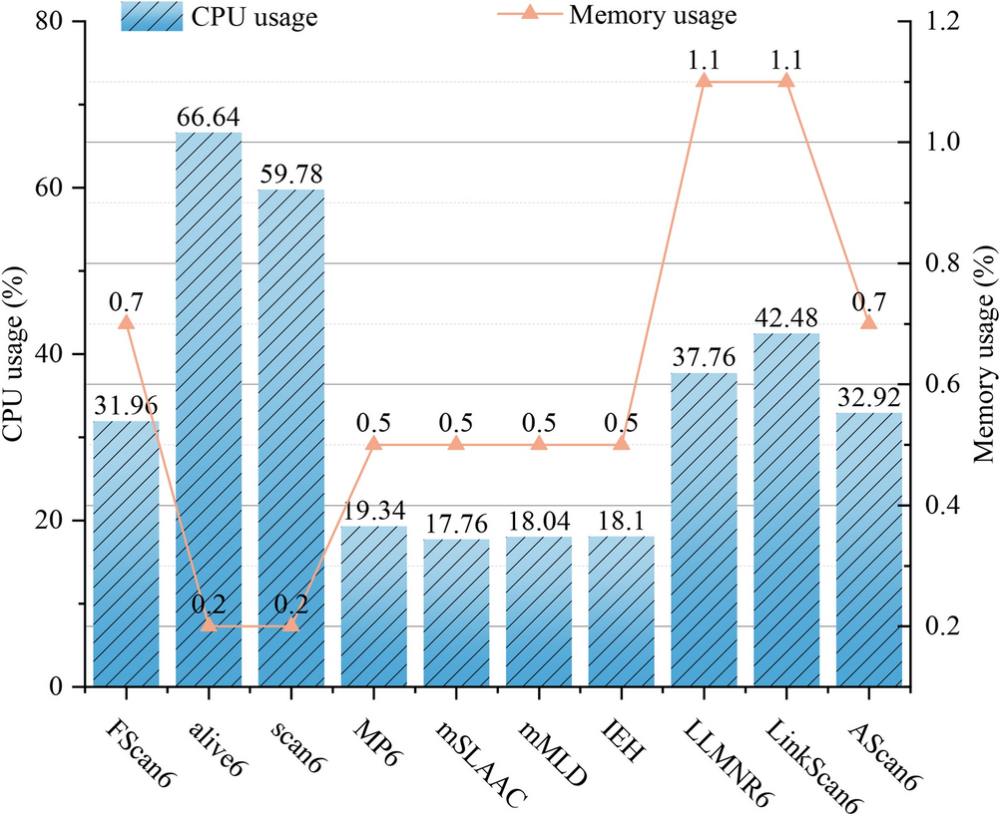
Usage of CPU and memory.

To assess the system resources required by FScan6 during scanning, this study used the htop tool to monitor CPU usage and memory usage of FScan6 and its comparison methods. The experimental results are presented in Fig. 11.

Fig. 11 illustrates the CPU and memory usage of the tools involved in the tests. Since address scanning is a network-intensive task, on-link address scanning tools generally have low memory demands, with memory usage typically remaining below 2%. In terms of CPU usage, the 4 address scanning scripts of Nmap require fewer CPU resources than FScan6, LLMNR6, LinkScan6, and AScan6 tools. This can be primarily attributed to the fact that 4 address scanning scripts of Nmap only require a single round of packet parsing, whereas tools like FScan6 necessitate multiple rounds of packet parsing, thereby increasing CPU consumption.

(2) The impact of FScan6 on network performance

To assess the impact of FScan6 on network performance during scanning, this study, inspired by Lin et al.^50^, run FScan6 under varying network loads while simultaneously sending ICMPv6 Echo Request messages to another host on-link. The RTT and packet loss rate between the request and response messages were measured. RTT serves as an indicator of network latency, with higher RTT values signaling a decline in network performance. On the other hand, the packet loss rate reflects the reliability of network communication, and an increase in packet loss directly affects the quality of communication, thereby influencing the number of addresses detected during scanning.

**Fig. 12:**
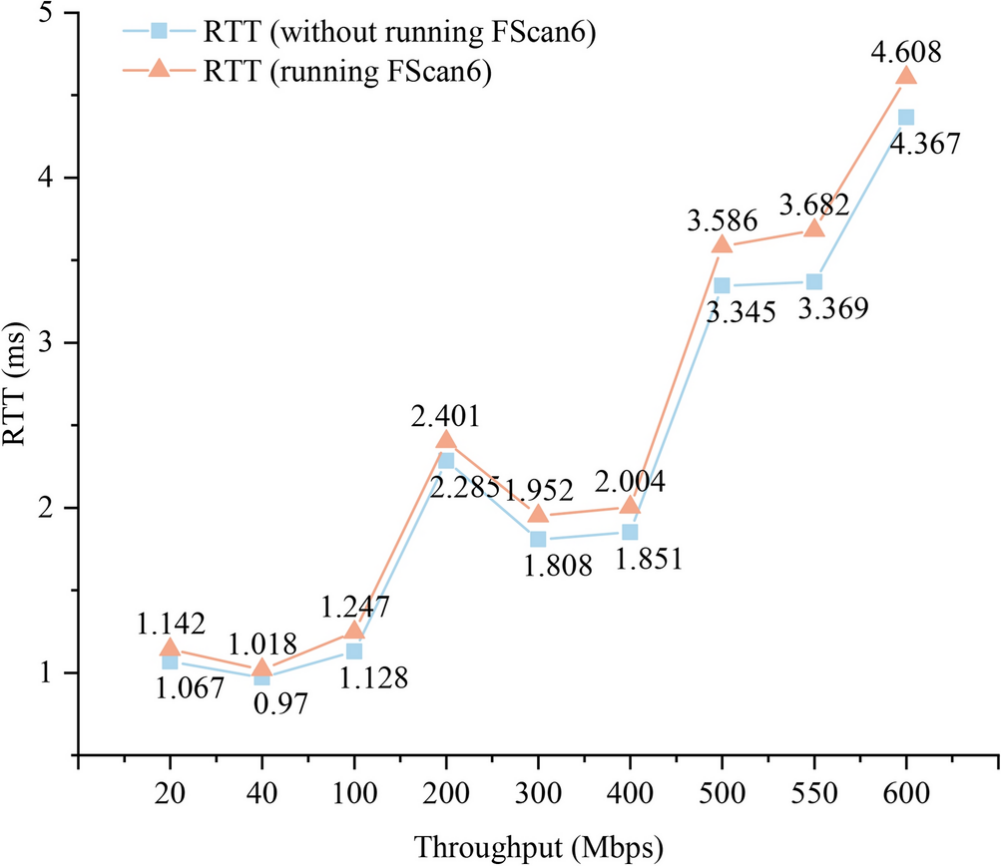
The impact of FScan6 on RTT.

Fig. 12 illustrates the impact of the FScan6 tool on RTT, while the impact on packet loss rate is presented in Table 8. As network load increases, the overall trend of RTT rises for both scenarios, when FScan6 is running and when it is not. When throughput is between 20-400 Mbps, the impact of FScan6 on RTT is minimal, with the maximum increase in RTT being only 0.153 ms, and the packet loss rate remaining at 0%. However, when throughput ranges from 500-600 Mbps, running FScan6 results in a maximum increase in RTT of 0.313 ms, with the packet loss rate still remaining at 0%. This is primarily attributed to FScan6’s ability to perform address scanning through an optional passive listening mode. Using this method, FScan6 generates and transmits only a minimal number of packets, thereby significantly reducing its impact on network performance.

**Table 8:** The impact of FScan6 on packet loss.

Throughput (Mbps)	20 (%)	40 (%)	100 (%)	200 (%)	300 (%)	400 (%)	500 (%)	550 (%)	600 (%)
Packet loss rate without FScan6	0	0	0	0	0	0	0	0	0
Packet loss rate with FScan6	0	0	0	0	0	0	0	0	0

Overall, FScan6 demonstrated a minimal impact on network performance, both under high and low network load conditions, with the effects remaining within acceptable limits. Furthermore, no packet loss was observed in any of the scenarios.

## Critical review of the proposed work

The proposed work represents a significant advancement, offering higher coverage of different OSs and greater completeness of scanning results compared to existing on-link IPv6 address scanning methods. However, due to the multiple stages of packet transmission, reception, and parsing involved in the proposed work, its scanning speed is somewhat slower than that of current solutions. Additionally, while the proposed method is technically robust, it raises ethical concerns regarding data privacy and the potential misuse of the detection results, which should be addressed appropriately.

To overcome these limitations, future work plans to leverage the Data Plane Development Kit to optimize the packet transmission and reception processes, thereby enhancing the scanning efficiency of the on-link IPv6 address scanning methods. Additionally, stricter privacy protection measures will be implemented to address ethical concerns more effectively.

## Conclusion

To quickly and completely obtain the GUAs and LLAs of active nodes within on-link IPv6 environment, and to minimize the impact on the network, an IPv6 address fast scanning method based on local domain name associations, which combines active scanning and passive listening, is proposed in this paper. It uses the association information to detect the IPv6 active addresses, suitable for IPv4/IPv6 dual-stack network environments.

The developed FScan6 tool can scan 26 mainstream versions of OSs (including 9 Windows versions, 10 Apple OS versions, and 7 Linux versions), quickly obtain all IPv6 GUAs and LLAs (71 IPv6 addresses in total), and continuously monitor and obtain most of the IPv6 addresses of subsequent new hosts joining the network. Compared to other on-link IPv6 address scanning methods, such as alive6, scan6, 4 address scanning scripts of Nmap, LinkScan6, LLMNR6, and AScan6, FScan6 demonstrates superior performance by identifying IPv6 addresses across the widest range of OS versions. Moreover, the number of IPv6 addresses discovered can be up to 4.18 times greater than those detected by the aforementioned scripts and tools.

In summary, the FScan6 tool possesses the highest completeness of scanning results, the highest coverage of different OSs, and low impact on network performance. It can provide a complete list of active hosts for the subsequent scanning of IPv6 network assets, the discovery of security vulnerabilities, and the optimization of network configurations. At the same time, to adapt to the scanning requirements in different environments, various modules of our development tool can be organically combined.

Next, the process of sending and receiving packets will be optimized by using the Data Plane Development Kit in future research to further improve the efficiency of IPv6 address scanning. Additionally, off-link IPv6 addresses will be discovered by leveraging other association information and the DNS servers on the Internet.

## Data Availability

The data that support the findings of this study are available from the corresponding author, upon reasonable request.
